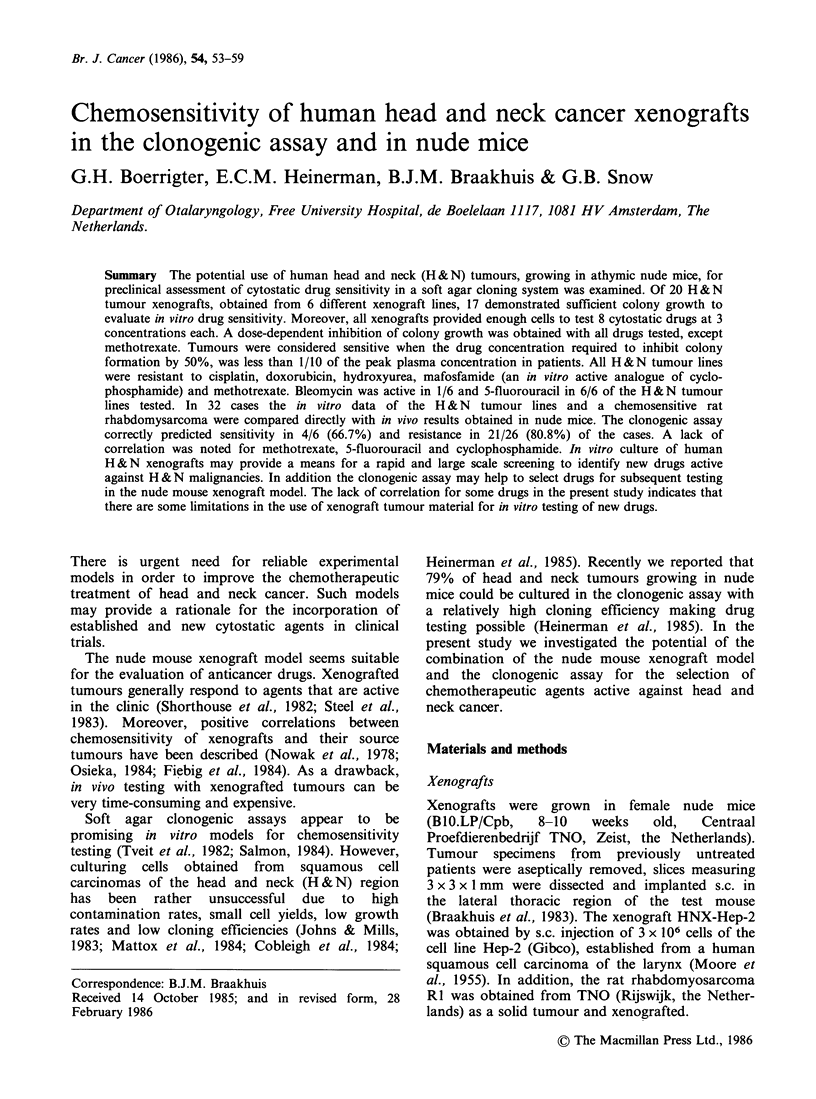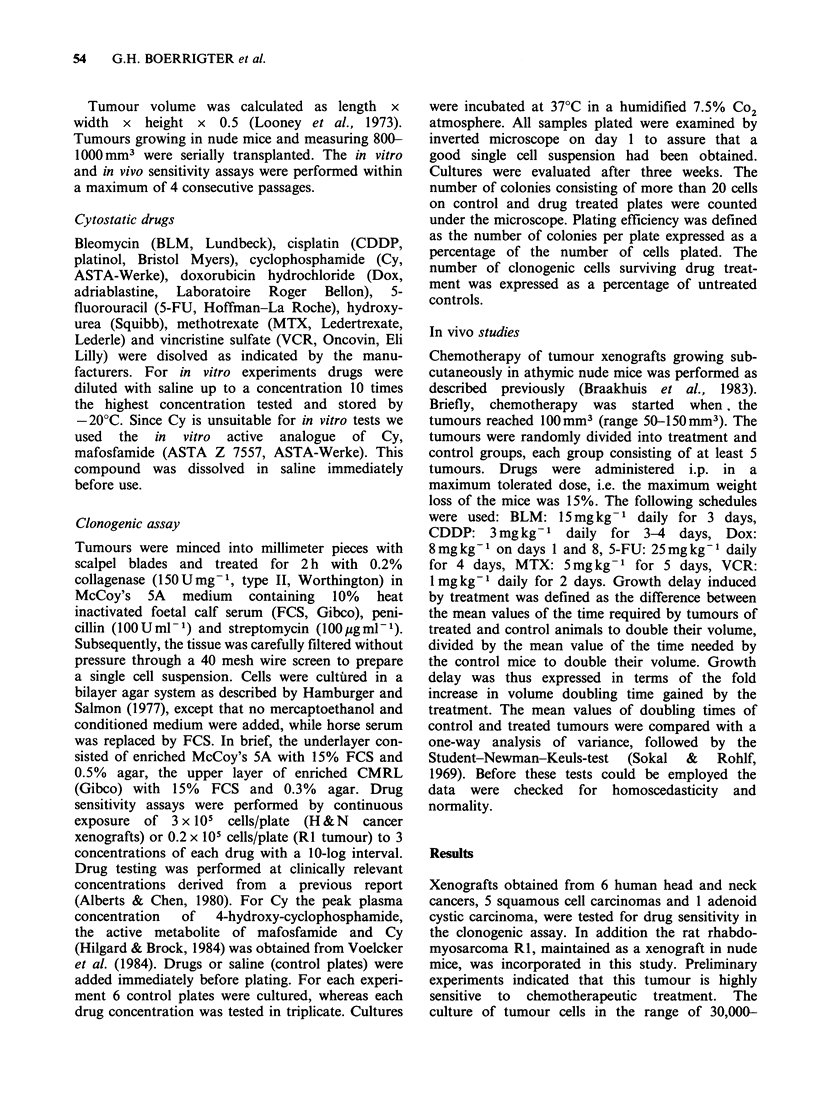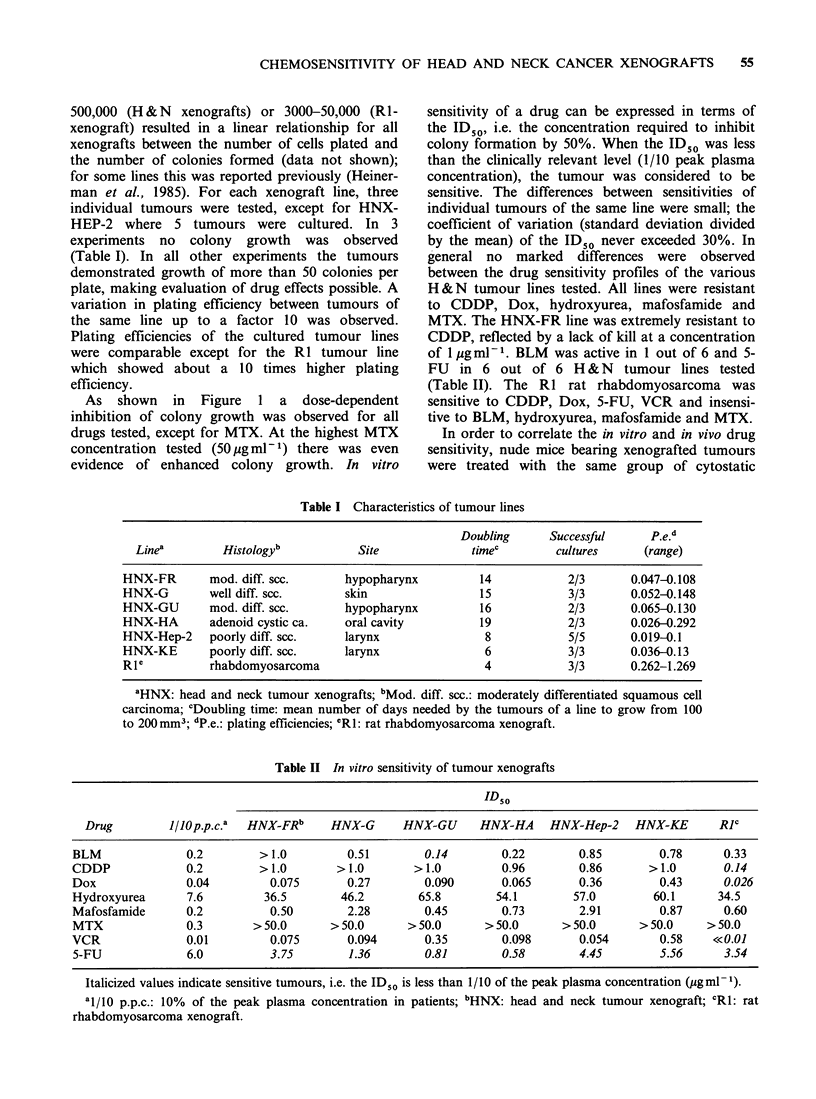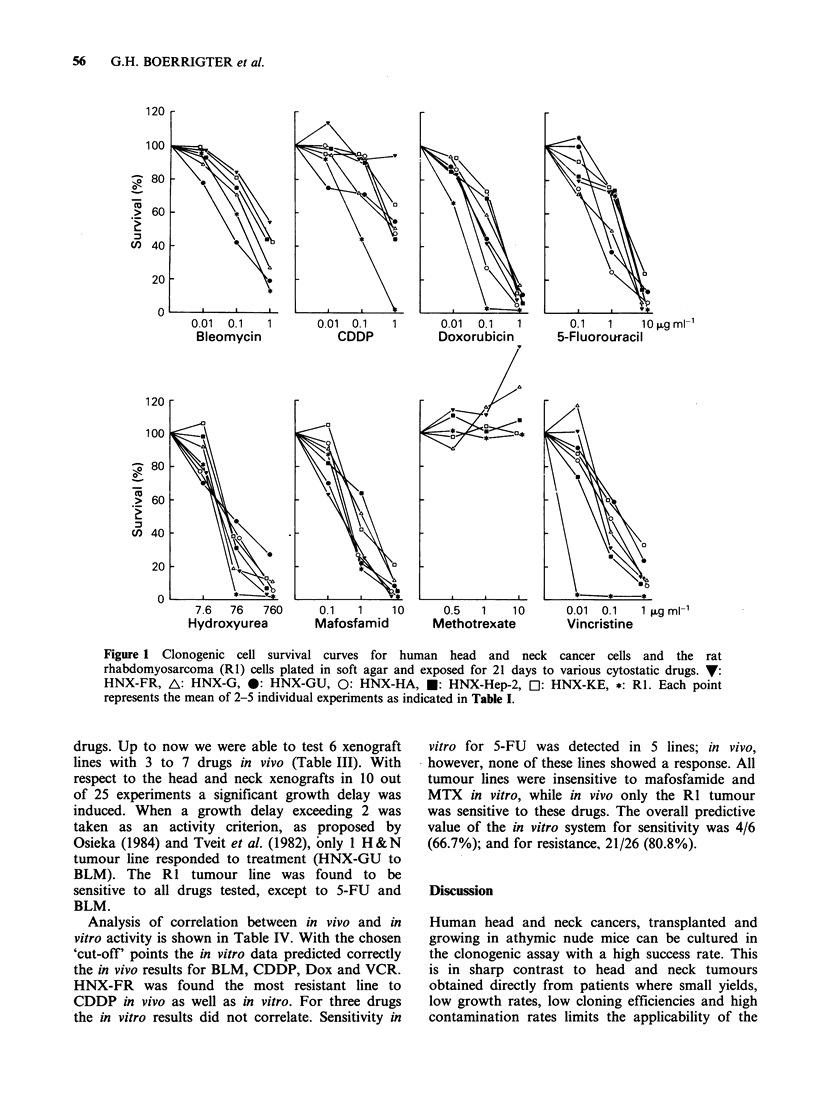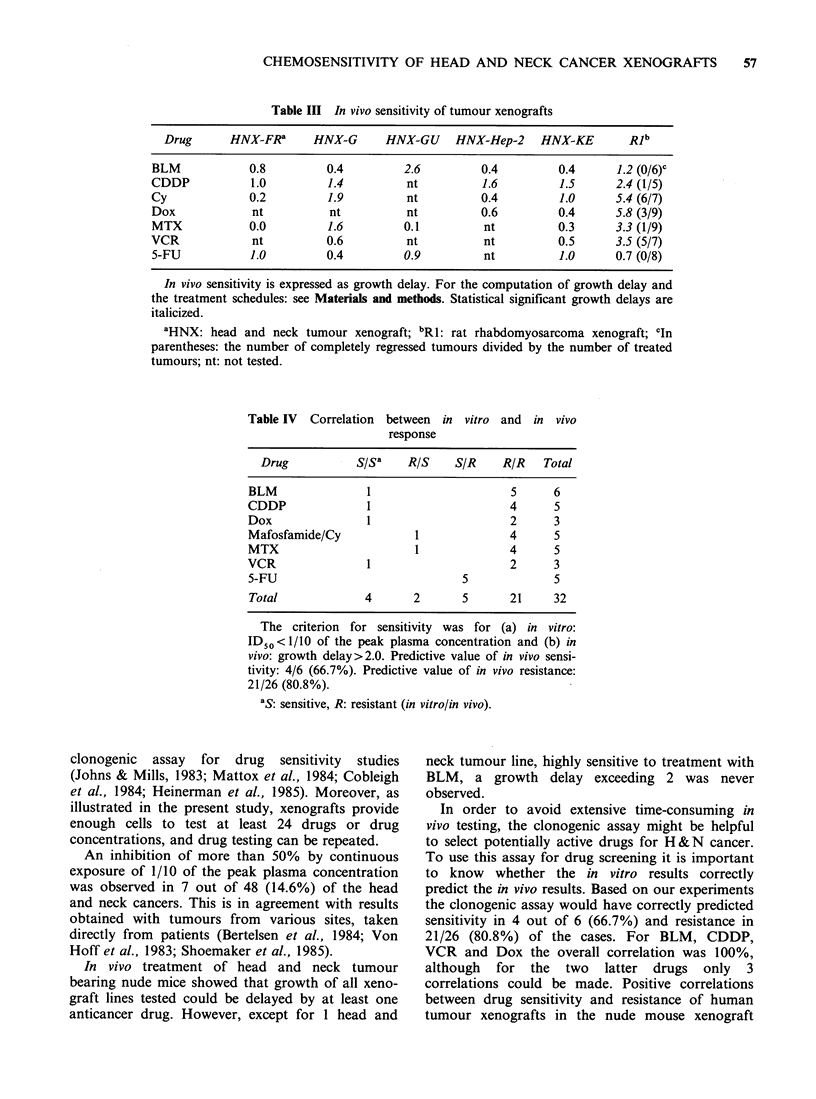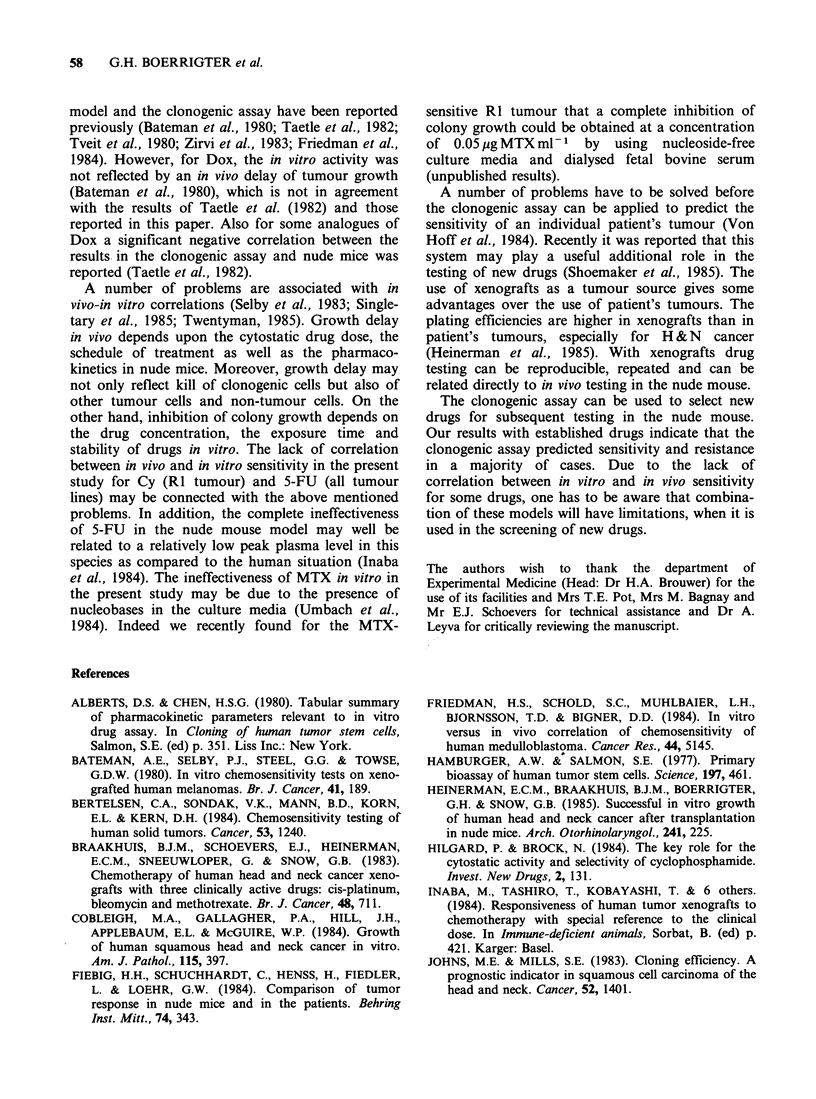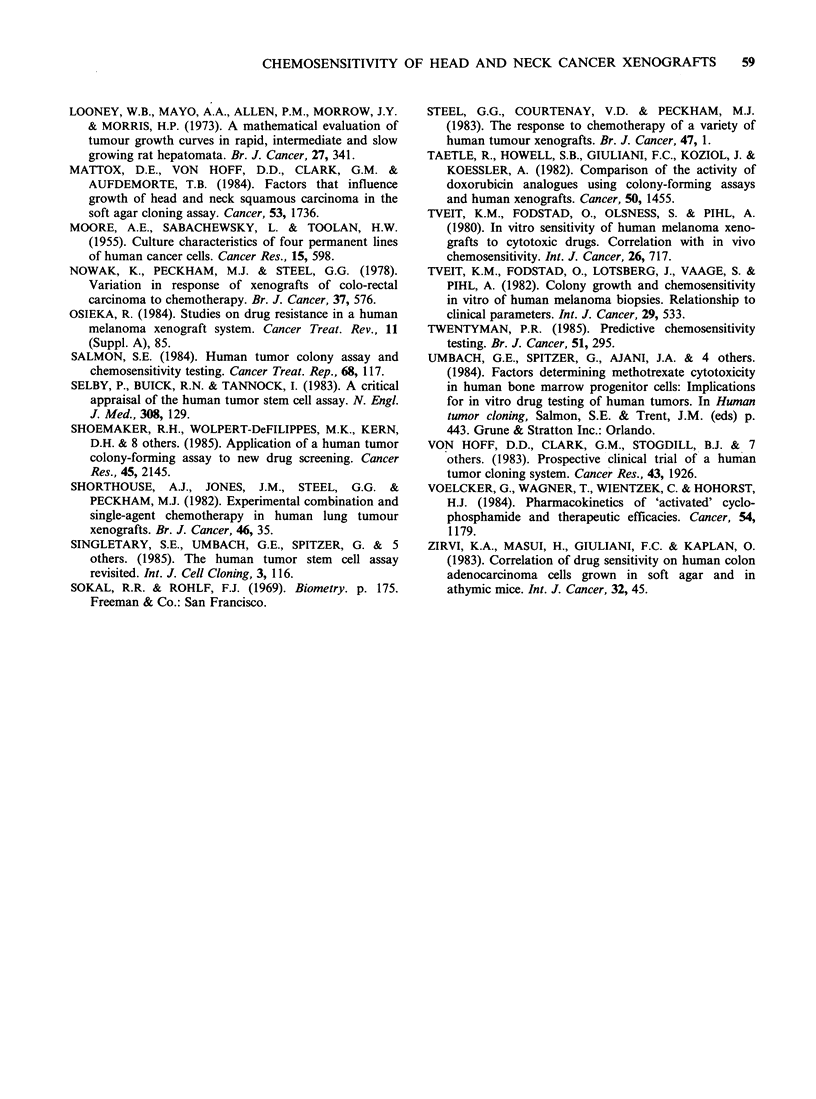# Chemosensitivity of human head and neck cancer xenografts in the clonogenic assay and in nude mice.

**DOI:** 10.1038/bjc.1986.151

**Published:** 1986-07

**Authors:** G. H. Boerrigter, E. C. Heinerman, B. J. Braakhuis, G. B. Snow

## Abstract

The potential use of human head and neck (H & N) tumours, growing in athymic nude mice, for preclinical assessment of cytostatic drug sensitivity in a soft agar cloning system was examined. Of 20 H & N tumour xenografts, obtained from 6 different xenograft lines, 17 demonstrated sufficient colony growth to evaluate in vitro drug sensitivity. Moreover, all xenografts provided enough cells to test 8 cytostatic drugs at 3 concentrations each. A dose-dependent inhibition of colony growth was obtained with all drugs tested, except methotrexate. Tumours were considered sensitive when the drug concentration required to inhibit colony formation by 50%, was less than 1/10 of the peak plasma concentration in patients. All H & N tumour lines were resistant to cisplatin, doxorubicin, hydroxyurea, mafosfamide (an in vitro active analogue of cyclophosphamide) and methotrexate. Bleomycin was active in 1/6 and 5-fluorouracil in 6/6 of the H & N tumour lines tested. In 32 cases the in vitro data of the H & N tumour lines and a chemosensitive rat rhabdomyosarcoma were compared directly with in vivo results obtained in nude mice. The clonogenic assay correctly predicted sensitivity in 4/6 (66.7%) and resistance in 21/26 (80.8%) of the cases. A lack of correlation was noted for methotrexate, 5-fluorouracil and cyclophosphamide. In vitro culture of human H & N xenografts may provide a means for a rapid and large scale screening to identify new drugs active against H & N malignancies. In addition the clonogenic assay may help to select drugs for subsequent testing in the nude mouse xenograft model. The lack of correlation for some drugs in the present study indicates that there are some limitations in the use of xenograft tumour material for in vitro testing of new drugs.


*Br. J. Cancer* (1986), **54**, 53–59

### Chemosensitivity of human head and neck cancer xenografts in the clonogenic assay and in nude mice

G.H. Boerrigter, E.C.M. Heinerman, B.J.M. Braakhuis & G.B. Snow

*Department of Otalaryngology, Free University Hospital, de Boelelaan 1117, 1081 HV Amsterdam, The Netherlands.*

**Summary** The potential use of human head and neck (H & N) tumours, growing in athymic nude mice, for preclinical assessment of cytostatic drug sensitivity in a soft agar cloning system was examined. Of 20 H & N tumour xenografts, obtained from 6 different xenograft lines, 17 demonstrated sufficient colony growth to evaluate *in vitro* drug sensitivity. Moreover, all xenografts provided enough cells to test 8 cytostatic drugs at 3 concentrations each. A dose-dependent inhibition of colony growth was obtained with all drugs tested, except methotrexate. Tumours were considered sensitive when the drug concentration required to inhibit colony formation by 50%, was less than 1/10 of the peak plasma concentration in patients. All H & N tumour lines were resistant to cisplatin, doxorubicin, hydroxyurea, mafosfamide (an *in vitro* active analogue of cyclophosphamide) and methotrexate. Bleomycin was active in 1/6 and 5-fluorouracil in 6/6 of the H & N tumour lines tested. In 32 cases the *in vitro* data of the H & N tumour lines and a chemosensitive rat rhabdomysarcoma were compared directly with *in vivo* results obtained in nude mice. The clonogenic assay correctly predicted sensitivity in 4/6 (66.7%) and resistance in 21/26 (80.8%) of the cases. A lack of correlation was noted for methotrexate, 5-fluorouracil and cyclophosphamide. *In vitro* culture of human H & N xenografts may provide a means for a rapid and large scale screening to identify new drugs active against H & N malignancies. In addition the clonogenic assay may help to select drugs for subsequent testing in the nude mouse xenograft model. The lack of correlation for some drugs in the present study indicates that there are some limitations in the use of xenograft tumour material for *in vitro* testing of new drugs.

There is urgent need for reliable experimental models in order to improve the chemotherapeutic treatment of head and neck cancer. Such models may provide a rationale for the incorporation of established and new cytostatic agents in clinical trials.

The nude mouse xenograft model seems suitable for the evaluation of anticancer drugs. Xenografted tumours generally respond to agents that are active in the clinic (Shorthouse *et al.*, 1982; Steel *et al.*, 1983). Moreover, positive correlations between chemosensitivity of xenografts and their source tumours have been described (Nowak *et al.*, 1978; Osieka, 1984; Fiebig *et al.*, 1984). As a drawback, *in vivo* testing with xenografted tumours can be very time-consuming and expensive.

Soft agar clonogenic assays appear to be promising *in vitro* models for chemosensitivity testing (Tveit *et al.*, 1982; Salmon, 1984). However, culturing cells obtained from squamous cell carcinomas of the head and neck (H & N) region has been rather unsuccessful due to high contamination rates, small cell yields, low growth rates and low cloning efficiencies (Johns & Mills, 1983; Mattox *et al.*, 1984; Cobleigh *et al.*, 1984; Heinerman *et al.*, 1985). Recently we reported that 79% of head and neck tumours growing in nude mice could be cultured in the clonogenic assay with a relatively high cloning efficiency making drug testing possible (Heinerman *et al.*, 1985). In the present study we investigated the potential of the combination of the nude mouse xenograft model and the clonogenic assay for the selection of chemotherapeutic agents active against head and neck cancer.

### Materials and methods

### *Xenografts*

Xenografts were grown in female nude mice (B10.LP/Cpb, 8–10 weeks old, Centraal Proefdierenbedrijf TNO, Zeist, the Netherlands). Tumour specimens from previously untreated patients were aseptically removed, slices measuring $3 \times 3 \times 1$ mm were dissected and implanted s.c. in the lateral thoracic region of the test mouse (Braakhuis *et al.*, 1983). The xenograft HNX-Hep-2 was obtained by s.c. injection of $3 \times 10^6$ cells of the cell line Hep-2 (Gibco), established from a human squamous cell carcinoma of the larynx (Moore *et al.*, 1955). In addition, the rat rhabdomyosarcoma R1 was obtained from TNO (Rijswijk, the Netherlands) as a solid tumour and xenografted.

Correspondence: B.J.M. Braakhuis
Received 14 October 1985; and in revised form, 28 February 1986

© The Macmillan Press Ltd., 1986

Tumour volume was calculated as length × width × height × 0.5 (Looney *et al.*, 1973). Tumours growing in nude mice and measuring 800–1000 mm$^3$ were serially transplanted. The *in vitro* and *in vivo* sensitivity assays were performed within a maximum of 4 consecutive passages.

*Cytostatic drugs*

Bleomycin (BLM, Lundbeck), cisplatin (CDDP, platinol, Bristol Myers), cyclophosphamide (Cy, ASTA-Werke), doxorubicin hydrochloride (Dox, adriablastine, Laboratoire Roger Bellon), 5-fluorouracil (5-FU, Hoffman–La Roche), hydroxyurea (Squibb), methotrexate (MTX, Ledertrexate, Lederle) and vincristine sulfate (VCR, Oncovin, Eli Lilly) were disolved as indicated by the manufacturers. For *in vitro* experiments drugs were diluted with saline up to a concentration 10 times the highest concentration tested and stored by −20°C. Since Cy is unsuitable for *in vitro* tests we used the *in vitro* active analogue of Cy, mafosfamide (ASTA Z 7557, ASTA-Werke). This compound was dissolved in saline immediately before use.

*Clonogenic assay*

Tumours were minced into millimeter pieces with scalpel blades and treated for 2 h with 0.2% collagenase (150 U mg$^{-1}$, type II, Worthington) in McCoy's 5A medium containing 10% heat inactivated foetal calf serum (FCS, Gibco), penicillin (100 U ml$^{-1}$) and streptomycin (100 $\mu$g ml$^{-1}$). Subsequently, the tissue was carefully filtered without pressure through a 40 mesh wire screen to prepare a single cell suspension. Cells were cultured in a bilayer agar system as described by Hamburger and Salmon (1977), except that no mercaptoethanol and conditioned medium were added, while horse serum was replaced by FCS. In brief, the underlayer consisted of enriched McCoy's 5A with 15% FCS and 0.5% agar, the upper layer of enriched CMRL (Gibco) with 15% FCS and 0.3% agar. Drug sensitivity assays were performed by continuous exposure of $3 \times 10^5$ cells/plate (H&N cancer xenografts) or $0.2 \times 10^5$ cells/plate (R1 tumour) to 3 concentrations of each drug with a 10-log interval. Drug testing was performed at clinically relevant concentrations derived from a previous report (Alberts & Chen, 1980). For Cy the peak plasma concentration of 4-hydroxy-cyclophosphamide, the active metabolite of mafosfamide and Cy (Hilgard & Brock, 1984) was obtained from Voelcker *et al.* (1984). Drugs or saline (control plates) were added immediately before plating. For each experiment 6 control plates were cultured, whereas each drug concentration was tested in triplicate. Cultures were incubated at 37°C in a humidified 7.5% $Co_2$ atmosphere. All samples plated were examined by inverted microscope on day 1 to assure that a good single cell suspension had been obtained. Cultures were evaluated after three weeks. The number of colonies consisting of more than 20 cells on control and drug treated plates were counted under the microscope. Plating efficiency was defined as the number of colonies per plate expressed as a percentage of the number of cells plated. The number of clonogenic cells surviving drug treatment was expressed as a percentage of untreated controls.

In vivo *studies*

Chemotherapy of tumour xenografts growing subcutaneously in athymic nude mice was performed as described previously (Braakhuis *et al.*, 1983). Briefly, chemotherapy was started when the tumours reached 100 mm$^3$ (range 50–150 mm$^3$). The tumours were randomly divided into treatment and control groups, each group consisting of at least 5 tumours. Drugs were administered i.p. in a maximum tolerated dose, i.e. the maximum weight loss of the mice was 15%. The following schedules were used: BLM: 15 mg kg$^{-1}$ daily for 3 days, CDDP: 3 mg kg$^{-1}$ daily for 3–4 days, Dox: 8 mg kg$^{-1}$ on days 1 and 8, 5-FU: 25 mg kg$^{-1}$ daily for 4 days, MTX: 5 mg kg$^{-1}$ for 5 days, VCR: 1 mg kg$^{-1}$ daily for 2 days. Growth delay induced by treatment was defined as the difference between the mean values of the time required by tumours of treated and control animals to double their volume, divided by the mean value of the time needed by the control mice to double their volume. Growth delay was thus expressed in terms of the fold increase in volume doubling time gained by the treatment. The mean values of doubling times of control and treated tumours were compared with a one-way analysis of variance, followed by the Student–Newman–Keuls-test (Sokal & Rohlf, 1969). Before these tests could be employed the data were checked for homoscedasticity and normality.

### Results

Xenografts obtained from 6 human head and neck cancers, 5 squamous cell carcinomas and 1 adenoid cystic carcinoma, were tested for drug sensitivity in the clonogenic assay. In addition the rat rhabdomyosarcoma R1, maintained as a xenograft in nude mice, was incorporated in this study. Preliminary experiments indicated that this tumour is highly sensitive to chemotherapeutic treatment. The culture of tumour cells in the range of 30,000–

500,000 (H&N xenografts) or 3000–50,000 (R1-xenograft) resulted in a linear relationship for all xenografts between the number of cells plated and the number of colonies formed (data not shown); for some lines this was reported previously (Heinerman *et al.,* 1985). For each xenograft line, three individual tumours were tested, except for HNX-HEP-2 where 5 tumours were cultured. In 3 experiments no colony growth was observed (Table I). In all other experiments the tumours demonstrated growth of more than 50 colonies per plate, making evaluation of drug effects possible. A variation in plating efficiency between tumours of the same line up to a factor 10 was observed. Plating efficiencies of the cultured tumour lines were comparable except for the R1 tumour line which showed about a 10 times higher plating efficiency.

As shown in Figure 1 a dose-dependent inhibition of colony growth was observed for all drugs tested, except for MTX. At the highest MTX concentration tested (50 $\mu g\,ml^{-1}$) there was even evidence of enhanced colony growth. *In vitro* sensitivity of a drug can be expressed in terms of the $ID_{50}$, i.e. the concentration required to inhibit colony formation by 50%. When the $ID_{50}$ was less than the clinically relevant level (1/10 peak plasma concentration), the tumour was considered to be sensitive. The differences between sensitivities of individual tumours of the same line were small; the coefficient of variation (standard deviation divided by the mean) of the $ID_{50}$ never exceeded 30%. In general no marked differences were observed between the drug sensitivity profiles of the various H&N tumour lines tested. All lines were resistant to CDDP, Dox, hydroxyurea, mafosfamide and MTX. The HNX-FR line was extremely resistant to CDDP, reflected by a lack of kill at a concentration of 1 $\mu g\,ml^{-1}$. BLM was active in 1 out of 6 and 5-FU in 6 out of 6 H&N tumour lines tested (Table II). The R1 rat rhabdomyosarcoma was sensitive to CDDP, Dox, 5-FU, VCR and insensitive to BLM, hydroxyurea, mafosfamide and MTX.

In order to correlate the *in vitro* and *in vivo* drug sensitivity, nude mice bearing xenografted tumours were treated with the same group of cytostatic

**Table I** Characteristics of tumour lines

| *Line*[a] | *Histology*[b] | *Site* | *Doubling time*[c] | *Successful cultures* | *P.e.*[d] *(range)* |
|---|---|---|---|---|---|
| HNX-FR | mod. diff. scc. | hypopharynx | 14 | 2/3 | 0.047–0.108 |
| HNX-G | well diff. scc. | skin | 15 | 3/3 | 0.052–0.148 |
| HNX-GU | mod. diff. scc. | hypopharynx | 16 | 2/3 | 0.065–0.130 |
| HNX-HA | adenoid cystic ca. | oral cavity | 19 | 2/3 | 0.026–0.292 |
| HNX-Hep-2 | poorly diff. scc. | larynx | 8 | 5/5 | 0.019–0.1 |
| HNX-KE | poorly diff. scc. | larynx | 6 | 3/3 | 0.036–0.13 |
| R1[e] | rhabdomyosarcoma | | 4 | 3/3 | 0.262–1.269 |

[a]HNX: head and neck tumour xenografts; [b]Mod. diff. scc.: moderately differentiated squamous cell carcinoma; [c]Doubling time: mean number of days needed by the tumours of a line to grow from 100 to 200 $mm^3$; [d]P.e.: plating efficiencies; [e]R1: rat rhabdomyosarcoma xenograft.

**Table II** *In vitro* sensitivity of tumour xenografts

| | | *$ID_{50}$* | | | | | | |
|---|---|---|---|---|---|---|---|---|
| *Drug* | *1/10 p.p.c.*[a] | *HNX-FR*[b] | *HNX-G* | *HNX-GU* | *HNX-HA* | *HNX-Hep-2* | *HNX-KE* | *R1*[c] |
| BLM | 0.2 | >1.0 | 0.51 | *0.14* | 0.22 | 0.85 | 0.78 | 0.33 |
| CDDP | 0.2 | >1.0 | >1.0 | >1.0 | 0.96 | 0.86 | >1.0 | *0.14* |
| Dox | 0.04 | 0.075 | 0.27 | 0.090 | 0.065 | 0.36 | 0.43 | *0.026* |
| Hydroxyurea | 7.6 | 36.5 | 46.2 | 65.8 | 54.1 | 57.0 | 60.1 | 34.5 |
| Mafosfamide | 0.2 | 0.50 | 2.28 | 0.45 | 0.73 | 2.91 | 0.87 | 0.60 |
| MTX | 0.3 | >50.0 | >50.0 | >50.0 | >50.0 | >50.0 | >50.0 | >50.0 |
| VCR | 0.01 | 0.075 | 0.094 | 0.35 | 0.098 | 0.054 | 0.58 | *≪0.01* |
| 5-FU | 6.0 | *3.75* | *1.36* | *0.81* | *0.58* | *4.45* | *5.56* | *3.54* |

Italicized values indicate sensitive tumours, i.e. the $ID_{50}$ is less than 1/10 of the peak plasma concentration ($\mu g\,ml^{-1}$).

[a]1/10 p.p.c.: 10% of the peak plasma concentration in patients; [b]HNX: head and neck tumour xenograft; [c]R1: rat rhabdomyosarcoma xenograft.

**Figure 1** Clonogenic cell survival curves for human head and neck cancer cells and the rat rhabdomyosarcoma (R1) cells plated in soft agar and exposed for 21 days to various cytostatic drugs. ▼: HNX-FR, △: HNX-G, ●: HNX-GU, ○: HNX-HA, ■: HNX-Hep-2, □: HNX-KE, *: R1. Each point represents the mean of 2–5 individual experiments as indicated in **Table I**.

drugs. Up to now we were able to test 6 xenograft lines with 3 to 7 drugs *in vivo* (Table III). With respect to the head and neck xenografts in 10 out of 25 experiments a significant growth delay was induced. When a growth delay exceeding 2 was taken as an activity criterion, as proposed by Osieka (1984) and Tveit *et al.* (1982), only 1 H&N tumour line responded to treatment (HNX-GU to BLM). The R1 tumour line was found to be sensitive to all drugs tested, except to 5-FU and BLM.

Analysis of correlation between *in vivo* and *in vitro* activity is shown in Table IV. With the chosen 'cut-off' points the *in vitro* data predicted correctly the *in vivo* results for BLM, CDDP, Dox and VCR. HNX-FR was found the most resistant line to CDDP *in vivo* as well as *in vitro*. For three drugs the *in vitro* results did not correlate. Sensitivity *in vitro* for 5-FU was detected in 5 lines; *in vivo*, however, none of these lines showed a response. All tumour lines were insensitive to mafosfamide and MTX *in vitro*, while *in vivo* only the R1 tumour was sensitive to these drugs. The overall predictive value of the *in vitro* system for sensitivity was 4/6 (66.7%); and for resistance, 21/26 (80.8%).

### Discussion

Human head and neck cancers, transplanted and growing in athymic nude mice can be cultured in the clonogenic assay with a high success rate. This is in sharp contrast to head and neck tumours obtained directly from patients where small yields, low growth rates, low cloning efficiencies and high contamination rates limits the applicability of the

**Table III** *In vivo* sensitivity of tumour xenografts

| *Drug* | *HNX-FR*[a] | *HNX-G* | *HNX-GU* | *HNX-Hep-2* | *HNX-KE* | *R1*[b] |
|---|---|---|---|---|---|---|
| BLM | 0.8 | 0.4 | *2.6* | 0.4 | 0.4 | *1.2* (0/6)[c] |
| CDDP | 1.0 | *1.4* | nt | *1.6* | *1.5* | *2.4* (1/5) |
| Cy | 0.2 | *1.9* | nt | 0.4 | *1.0* | *5.4* (6/7) |
| Dox | nt | nt | nt | 0.6 | 0.4 | *5.8* (3/9) |
| MTX | 0.0 | *1.6* | 0.1 | nt | 0.3 | *3.3* (1/9) |
| VCR | nt | 0.6 | nt | nt | 0.5 | *3.5* (5/7) |
| 5-FU | *1.0* | 0.4 | *0.9* | nt | *1.0* | 0.7 (0/8) |

*In vivo* sensitivity is expressed as growth delay. For the computation of growth delay and the treatment schedules: see **Materials and methods**. Statistical significant growth delays are italicized.

[a]HNX: head and neck tumour xenograft; [b]R1: rat rhabdomyosarcoma xenograft; [c]In parentheses: the number of completely regressed tumours divided by the number of treated tumours; nt: not tested.

**Table IV** Correlation between *in vitro* and *in vivo* response

| *Drug* | *S/S*[a] | *R/S* | *S/R* | *R/R* | *Total* |
|---|---|---|---|---|---|
| BLM | 1 | | | 5 | 6 |
| CDDP | 1 | | | 4 | 5 |
| Dox | 1 | | | 2 | 3 |
| Mafosfamide/Cy | | 1 | | 4 | 5 |
| MTX | | 1 | | 4 | 5 |
| VCR | 1 | | | 2 | 3 |
| 5-FU | | | 5 | | 5 |
| *Total* | 4 | 2 | 5 | 21 | 32 |

The criterion for sensitivity was for (a) *in vitro*: $ID_{50} < 1/10$ of the peak plasma concentration and (b) *in vivo*: growth delay > 2.0. Predictive value of *in vivo* sensitivity: 4/6 (66.7%). Predictive value of *in vivo* resistance: 21/26 (80.8%).

[a]*S*: sensitive, *R*: resistant (*in vitro*/*in vivo*).

clonogenic assay for drug sensitivity studies (Johns & Mills, 1983; Mattox *et al.*, 1984; Cobleigh *et al.*, 1984; Heinerman *et al.*, 1985). Moreover, as illustrated in the present study, xenografts provide enough cells to test at least 24 drugs or drug concentrations, and drug testing can be repeated.

An inhibition of more than 50% by continuous exposure of 1/10 of the peak plasma concentration was observed in 7 out of 48 (14.6%) of the head and neck cancers. This is in agreement with results obtained with tumours from various sites, taken directly from patients (Bertelsen *et al.*, 1984; Von Hoff *et al.*, 1983; Shoemaker *et al.*, 1985).

*In vivo* treatment of head and neck tumour bearing nude mice showed that growth of all xenograft lines tested could be delayed by at least one anticancer drug. However, except for 1 head and neck tumour line, highly sensitive to treatment with BLM, a growth delay exceeding 2 was never observed.

In order to avoid extensive time-consuming *in vivo* testing, the clonogenic assay might be helpful to select potentially active drugs for H & N cancer. To use this assay for drug screening it is important to know whether the *in vitro* results correctly predict the *in vivo* results. Based on our experiments the clonogenic assay would have correctly predicted sensitivity in 4 out of 6 (66.7%) and resistance in 21/26 (80.8%) of the cases. For BLM, CDDP, VCR and Dox the overall correlation was 100%, although for the two latter drugs only 3 correlations could be made. Positive correlations between drug sensitivity and resistance of human tumour xenografts in the nude mouse xenograft model and the clonogenic assay have been reported previously (Bateman *et al.*, 1980; Taetle *et al.*, 1982; Tveit *et al.*, 1980; Zirvi *et al.*, 1983; Friedman *et al.*, 1984). However, for Dox, the *in vitro* activity was not reflected by an *in vivo* delay of tumour growth (Bateman *et al.*, 1980), which is not in agreement with the results of Taetle *et al.* (1982) and those reported in this paper. Also for some analogues of Dox a significant negative correlation between the results in the clonogenic assay and nude mice was reported (Taetle *et al.*, 1982).

A number of problems are associated with *in vivo-in vitro* correlations (Selby *et al.*, 1983; Singletary *et al.*, 1985; Twentyman, 1985). Growth delay *in vivo* depends upon the cytostatic drug dose, the schedule of treatment as well as the pharmacokinetics in nude mice. Moreover, growth delay may not only reflect kill of clonogenic cells but also of other tumour cells and non-tumour cells. On the other hand, inhibition of colony growth depends on the drug concentration, the exposure time and stability of drugs *in vitro*. The lack of correlation between *in vivo* and *in vitro* sensitivity in the present study for Cy (R1 tumour) and 5-FU (all tumour lines) may be connected with the above mentioned problems. In addition, the complete ineffectiveness of 5-FU in the nude mouse model may well be related to a relatively low peak plasma level in this species as compared to the human situation (Inaba *et al.*, 1984). The ineffectiveness of MTX *in vitro* in the present study may be due to the presence of nucleobases in the culture media (Umbach *et al.*, 1984). Indeed we recently found for the MTX-sensitive R1 tumour that a complete inhibition of colony growth could be obtained at a concentration of $0.05\,\mu g\,MTX\,ml^{-1}$ by using nucleoside-free culture media and dialysed fetal bovine serum (unpublished results).

A number of problems have to be solved before the clonogenic assay can be applied to predict the sensitivity of an individual patient's tumour (Von Hoff *et al.*, 1984). Recently it was reported that this system may play a useful additional role in the testing of new drugs (Shoemaker *et al.*, 1985). The use of xenografts as a tumour source gives some advantages over the use of patient's tumours. The plating efficiencies are higher in xenografts than in patient's tumours, especially for H&N cancer (Heinerman *et al.*, 1985). With xenografts drug testing can be reproducible, repeated and can be related directly to *in vivo* testing in the nude mouse.

The clonogenic assay can be used to select new drugs for subsequent testing in the nude mouse. Our results with established drugs indicate that the clonogenic assay predicted sensitivity and resistance in a majority of cases. Due to the lack of correlation between *in vitro* and *in vivo* sensitivity for some drugs, one has to be aware that combination of these models will have limitations, when it is used in the screening of new drugs.

The authors wish to thank the department of Experimental Medicine (Head: Dr H.A. Brouwer) for the use of its facilities and Mrs T.E. Pot, Mrs M. Bagnay and Mr E.J. Schoevers for technical assistance and Dr A. Leyva for critically reviewing the manuscript.

### References

ALBERTS, D.S. & CHEN, H.S.G. (1980). Tabular summary of pharmacokinetic parameters relevant to in vitro drug assay. In *Cloning of human tumor stem cells*, Salmon, S.E. (ed) p. 351. Liss Inc.: New York.

BATEMAN, A.E., SELBY, P.J., STEEL, G.G. & TOWSE, G.D.W. (1980). In vitro chemosensitivity tests on xenografted human melanomas. *Br. J. Cancer*, **41**, 189.

BERTELSEN, C.A., SONDAK, V.K., MANN, B.D., KORN, E.L. & KERN, D.H. (1984). Chemosensitivity testing of human solid tumors. *Cancer*, **53**, 1240.

BRAAKHUIS, B.J.M., SCHOEVERS, E.J., HEINERMAN, E.C.M., SNEEUWLOPER, G. & SNOW, G.B. (1983). Chemotherapy of human head and neck cancer xenografts with three clinically active drugs: cis-platinum, bleomycin and methotrexate. *Br. J. Cancer*, **48**, 711.

COBLEIGH, M.A., GALLAGHER, P.A., HILL, J.H., APPLEBAUM, E.L. & McGUIRE, W.P. (1984). Growth of human squamous head and neck cancer in vitro. *Am. J. Pathol.*, **115**, 397.

FIEBIG, H.H., SCHUCHHARDT, C., HENSS, H., FIEDLER, L. & LOEHR, G.W. (1984). Comparison of tumor response in nude mice and in the patients. *Behring Inst. Mitt.*, **74**, 343.

FRIEDMAN, H.S., SCHOLD, S.C., MUHLBAIER, L.H., BJORNSSON, T.D. & BIGNER, D.D. (1984). In vitro versus in vivo correlation of chemosensitivity of human medulloblastoma. *Cancer Res.*, **44**, 5145.

HAMBURGER, A.W. & SALMON, S.E. (1977). Primary bioassay of human tumor stem cells. *Science*, **197**, 461.

HEINERMAN, E.C.M., BRAAKHUIS, B.J.M., BOERRIGTER, G.H. & SNOW, G.B. (1985). Successful in vitro growth of human head and neck cancer after transplantation in nude mice. *Arch. Otorhinolaryngol.*, **241**, 225.

HILGARD, P. & BROCK, N. (1984). The key role for the cytostatic activity and selectivity of cyclophosphamide. *Invest. New Drugs*, **2**, 131.

INABA, M., TASHIRO, T., KOBAYASHI, T. & 6 others. (1984). Responsiveness of human tumor xenografts to chemotherapy with special reference to the clinical dose. In *Immune-deficient animals*, Sorbat, B. (ed) p. 421. Karger: Basel.

JOHNS, M.E. & MILLS, S.E. (1983). Cloning efficiency. A prognostic indicator in squamous cell carcinoma of the head and neck. *Cancer*, **52**, 1401.

LOONEY, W.B., MAYO, A.A., ALLEN, P.M., MORROW, J.Y. & MORRIS, H.P. (1973). A mathematical evaluation of tumour growth curves in rapid, intermediate and slow growing rat hepatomata. *Br. J. Cancer*, **27**, 341.

MATTOX, D.E., VON HOFF, D.D., CLARK, G.M. & AUFDEMORTE, T.B. (1984). Factors that influence growth of head and neck squamous carcinoma in the soft agar cloning assay. *Cancer*, **53**, 1736.

MOORE, A.E., SABACHEWSKY, L. & TOOLAN, H.W. (1955). Culture characteristics of four permanent lines of human cancer cells. *Cancer Res.*, **15**, 598.

NOWAK, K., PECKHAM, M.J. & STEEL, G.G. (1978). Variation in response of xenografts of colo-rectal carcinoma to chemotherapy. *Br. J. Cancer*, **37**, 576.

OSIEKA, R. (1984). Studies on drug resistance in a human melanoma xenograft system. *Cancer Treat. Rev.*, **11** (Suppl. A), 85.

SALMON, S.E. (1984). Human tumor colony assay and chemosensitivity testing. *Cancer Treat. Rep.*, **68**, 117.

SELBY, P., BUICK, R.N. & TANNOCK, I. (1983). A critical appraisal of the human tumor stem cell assay. *N. Engl. J. Med.*, **308**, 129.

SHOEMAKER, R.H., WOLPERT-DeFILIPPES, M.K., KERN, D.H. & 8 others. (1985). Application of a human tumor colony-forming assay to new drug screening. *Cancer Res.*, **45**, 2145.

SHORTHOUSE, A.J., JONES, J.M., STEEL, G.G. & PECKHAM, M.J. (1982). Experimental combination and single-agent chemotherapy in human lung tumour xenografts. *Br. J. Cancer*, **46**, 35.

SINGLETARY, S.E., UMBACH, G.E., SPITZER, G. & 5 others. (1985). The human tumor stem cell assay revisited. *Int. J. Cell Cloning*, **3**, 116.

SOKAL, R.R. & ROHLF, F.J. (1969). *Biometry*. p. 175. Freeman & Co.: San Francisco.

STEEL, G.G., COURTENAY, V.D. & PECKHAM, M.J. (1983). The response to chemotherapy of a variety of human tumour xenografts. *Br. J. Cancer*, **47**, 1.

TAETLE, R., HOWELL, S.B., GIULIANI, F.C., KOZIOL, J. & KOESSLER, A. (1982). Comparison of the activity of doxorubicin analogues using colony-forming assays and human xenografts. *Cancer*, **50**, 1455.

TVEIT, K.M., FODSTAD, O., OLSNESS, S. & PIHL, A. (1980). In vitro sensitivity of human melanoma xenografts to cytotoxic drugs. Correlation with in vivo chemosensitivity. *Int. J. Cancer*, **26**, 717.

TVEIT, K.M., FODSTAD, O., LOTSBERG, J., VAAGE, S. & PIHL, A. (1982). Colony growth and chemosensitivity in vitro of human melanoma biopsies. Relationship to clinical parameters. *Int. J. Cancer*, **29**, 533.

TWENTYMAN, P.R. (1985). Predictive chemosensitivity testing. *Br. J. Cancer*, **51**, 295.

UMBACH, G.E., SPITZER, G., AJANI, J.A. & 4 others. (1984). Factors determining methotrexate cytotoxicity in human bone marrow progenitor cells: Implications for in vitro drug testing of human tumors. In *Human tumor cloning*, Salmon, S.E. & Trent, J.M. (eds) p. 443. Grune & Stratton Inc.: Orlando.

VON HOFF, D.D., CLARK, G.M., STOGDILL, B.J. & 7 others. (1983). Prospective clinical trial of a human tumor cloning system. *Cancer Res.*, **43**, 1926.

VOELCKER, G., WAGNER, T., WIENTZEK, C. & HOHORST, H.J. (1984). Pharmacokinetics of 'activated' cyclophosphamide and therapeutic efficacies. *Cancer*, **54**, 1179.

ZIRVI, K.A., MASUI, H., GIULIANI, F.C. & KAPLAN, O. (1983). Correlation of drug sensitivity on human colon adenocarcinoma cells grown in soft agar and in athymic mice. *Int. J. Cancer*, **32**, 45.